# A Morphometric Analysis of the *Santolina chamaecyparissus* Complex (Asteraceae)

**DOI:** 10.3390/plants11243458

**Published:** 2022-12-09

**Authors:** Antonio Giacò, Paola De Giorgi, Giovanni Astuti, Paolo Caputo, Miguel Serrano, Rodrigo Carballal, Llorenç Sáez, Gianluigi Bacchetta, Lorenzo Peruzzi

**Affiliations:** 1 PLANTSEED Lab, Department of Biology, University of Pisa, 56126 Pisa, Italy; 2 Botanic Garden and Museum, University of Pisa, 56126 Pisa, Italy; 3 Department of Biology, University of Naples Federico II, 80100 Naples, Italy; 4 Department of Botany, Faculty of Pharmacy, University of Santiago de Compostela, 15782 A Corunha, Spain; 5 Department BABVE, Faculty of Biosciences, Autonomous University of Barcelona, 08193 Bellaterra, Spain; 6 Centre for Conservation of Biodiversity (CCB), Department of Life and Environmental Sciences, University of Cagliari, 09123 Cagliari, Italy

**Keywords:** Mediterranean Basin, Anthemideae, endemism, morphometry, Random Forest

## Abstract

The genus *Santolina* (Asteraceae, Anthemideae) includes 26 species of aromatic evergreen shrubs endemic to the western Mediterranean Basin. *Santolina* is widely used as ornamental plant, in xerigardening, and in ethnobotany. The *Santolina chamaecyparissus* complex, including about half of the known species diversity, has been properly investigated on systematic and taxonomic grounds only recently, and a complete morphometric study is still missing. Here we provide a morphometric characterization and comparison of all the 14 species of this complex, using both univariate and multivariate analyses. Our results suggest that species of this complex can be distinguished using combinations of quantitative and qualitative character-states, mostly related to the leaf morphology. The analysis of *S. villosa*, a tetraploid/hexaploid Spanish endemic, showed that the two cytotypes cannot be safely identified based on morphology. Coupling this evidence with available phylogenetic information, we conclude that there is no reason to split the two cytotypes of *S. villosa* in two distinct taxa. An identification key for all the species of the complex is presented.

## 1. Introduction

*Santolina* L. (Anthemideae) is a genus of evergreen shrubs endemic to the western portion of the Mediterranean Basin [1]. Most species occur under Mediterranean bioclimate, usually on calcareous substrates [2,3,4]. Due to their ability to tolerate periods of strong drought, some species, and in particular *S. chamaecyparissus* L., are cultivated as ornamental plants and in xerigardening [5]. In addition, most species are known for their traditional ethnobotanical uses. For instance, the inflorescences of *S. chamaecyparissus*, *S. oblongifolia* Boiss., and *S. rosmarinifolia* L. were used for their anti-inflammatory effects [6], whereas aerial parts of *S. corsica* Jord. & Fourr., *S. ericoides* Poir., and *S. etrusca* (Lacaita) Marchi & D’Amato were used as vermifuge and antiparasitic [7,8]. The ethnobotanical importance of *Santolina* has stimulated in the last decades research concerning the biological properties and the phytochemical composition. Indeed, phytochemical studies discovered the presence of several compounds, such as terpenoids, chrysanthemane monoterpenoids, flavonoids, and coumarins, that are known for their effects on human health [9,10,11,12,13,14]. However, while the literature concerning the phytochemistry of *Santolina* was proliferating [10,15,16,17,18,19,20], the systematic knowledge of this genus has remained fragmentary and incomplete until recent years. Important contributions to the systematics and taxonomy of *Santolina* were provided by Carbajal and collaborators [4,21] for the *S. rosmarinifolia* complex, whose species mostly occur in the Iberian Peninsula, and by Giacò and collaborators for the *S. chamaecyparissus* complex, more widely distributed across the western Mediterranean Basin. As regards the latter, a nomenclatural revision [1] and a karyomorphological study [22] raised several taxonomic issues that have been later clarified using integrated taxonomic approaches. In particular, De Giorgi and collaborators [23] focused on polyploid *Santolina* populations from Corsica and Sardinia, Giacò and collaborators [24] on diploid continental Italian species, while Giacò and collaborators [25] untangled the systematic relationships of diploid populations occurring in southern France and north-eastern Spain. *Santolina insularis* (Gennari ex Fiori) Arrigoni has been synonymized with *S. corsica* [23], whereas new taxa have been recognized in France and Spain [25]: *S. intricata* Jord. & Fourr. and three allopatric subspecies within *S. decumbens* Mill. However, several taxa of the complex have not yet been properly studied, and an overall quantitative morphological analysis is still lacking. In addition, an important gap of knowledge concerns the evaluation of possible taxonomic distinction of the two cytotypes of *S. villosa* Mill., a tetraploid (2*n* = 4*x* = 36) and hexaploid (2*n* = 6*x* = 54) species that is endemic to central-eastern and southern Spain [22].

Accordingly, the aims of this study are (a) to quantitatively assess whether the two cytotypes of *S. villosa* can be distinguished on morphometric grounds, (b) to carry out an exhaustive univariate and multivariate morphometric analysis of the complex including all the 14 recognized species, and (c) to build an identification key.

## 2. Results

### 2.1. Morphometrics of the Two Cytotypes of S. villosa

**Figure 1:**
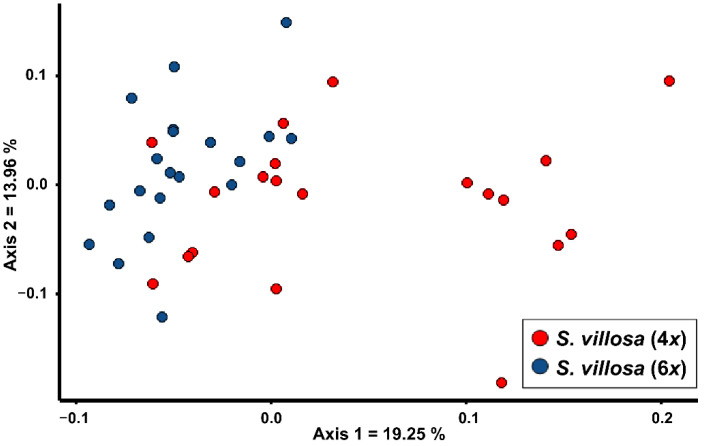
PCoA based on Gower distance showing the morphological relationships between the two cytotypes of *Santolina villosa*, a polyploid species endemic to central-eastern and southern Spain.

In Figure 1, a PCoA showing the two cytotypes of *S. villosa* is reported. The first two axes explain 33.21% of the morphological variability. The tetraploid population shows a wide morphological variability on the first axis, and partially overlaps with the hexaploid population on the left side of the graph.

The two populations significantly differ for eight quantitative character-states (Table 1). However, their Cohen’s d values are always <1.2, showing remarkable overlaps. In Table S1, the mean values ± standard deviation of each quantitative character is reported for each population, included the two studied populations/cytotypes of *S. villosa*. Conversely, no qualitative character shows significant differences. Assuming the two cytotypes as *a priori* groups, Random Forest returned a low value of overall correct classification (68.4%), further confirming the high morphological overlap.

### 2.2. Morphometrics of the Whole S. chamaecyparissus Complex

Random Forest returned a value of overall mean correct classification of 89.2% (Table 2), considering all the 14 species as *a priori* groups. *Santolina ericoides* and *S. pinnata* are correctly classified in 100% of cases. Conversely, *S. vedranensis* shows the lowest value of mean correct classification (59.9%), since it is confused mostly with *S. corsica* (22.9%) and *S. decumbens* (7.3%). Except for *S. intricata* (68.7%), *S. virens* (69.5%), and *S. decumbens* (81.8%), other species are well classified (>90%) by the algorithm.

**Figure 2:**
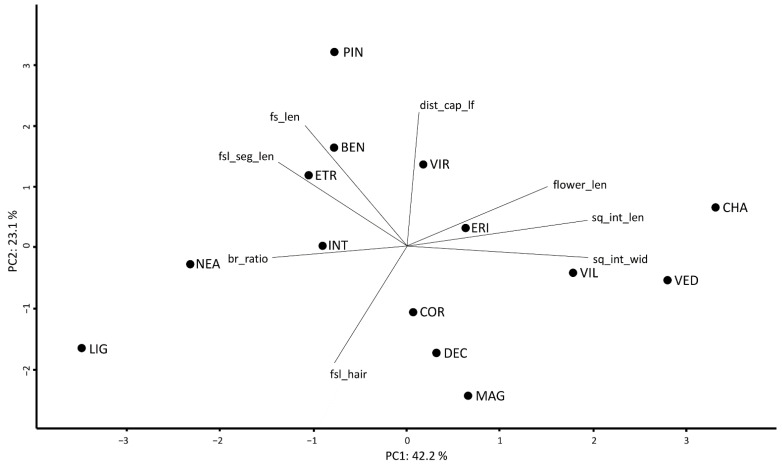
The morphometric analysis of the *S. chamaecyparissus* complex. PCA based on eight non-correlated variables, considering the mean values scored by each species. BEN = *S. benthamiana*, CHA = *S. chamaecyparissus*, COR = *S. corsica*, DEC = *S. decumbens*, ERI = *S. ericoides*, ETR = *S. etrusca*, INT = *S. intricata*, LIG = *S. ligustica*, MAG = *S. magonica*, NEA = *S. neapolitana*, PIN = *S. pinnata*, VED = *S. vedranensis*, VIL = *S. villosa*, and VIR = *S. virens*. br_ratio = ratio between the highest ramification of the flowering stem and fs_len, dist_cap_lf = distance between the highest leaf on the stem and the floral head (mm), flower_len = length of the floral tube (mm), fs_len = length of the flowering stem (cm), fsl_hair = degree of tomentosity of the flowering stem leaf segment (%), fsl_seg_len = length of the segment of the flowering stem leaf (mm), sq_int_len = length of the internal involucral bract (mm), and sq_int_wid = width of the internal involucral bract (mm).

By plotting the first two axes of a PCA based on the mean values of eight non-correlated characters (65.3% of the variance explained), the overall morphological relationships among species are highlighted (Figure 2).

In Table 3, the mean values ± standard deviation for each species and for each quantitative character are reported, whereas the same information is reported at population level in Table S1. In Table S2, the number of significantly different quantitative character-states showing Cohen’s d > 1.2 and the number of significantly different qualitative character-states are reported for each pair of species. The pair *S. chamaecyparissus* vs. *S. etrusca* shows the highest number of significantly different character-states (26 quantitative + 5 qualitative), whereas the pairs *S. benthamiana* vs. *S. intricata*, *S. decumbens* vs. *S. villosa*, and *S. ericoides* vs. *S. virens* show the lowest number (5 + 2, 2 + 5, and 3 + 4, respectively).

In Table S3, the quantitative characters that significantly differ with Cohen’s d > 1.2 and the significantly different qualitative character-states for each pair of species are reported. The quantitative character occurring with the highest frequency in the pairwise comparisons (69 times in Table S3) is the tomentosity of the flowering stems (fs_hair). The following nine characters showing high frequency (63 to 49 times) are still all related to the leaf morphology. The character with the lowest frequency (four times) is the length of the external involucral bract (sq_ext_len). Overall, quantitative characters related to the capitula morphology are less frequently represented than the characters related to the leaf morphology. The qualitative character occurring with the highest frequency (70 times in Table S3) is the tomentosity of the internal involucral bract (sq_int_hair). Conversely, the qualitative character with the lowest frequency (35 times in Table S3) is the colour of the flowers (fl_col).

## 3. Discussion

Our analyses showed that it is almost impossible to distinguish the two cytotypes of *S. villosa*. Albeit the tetraploids exhibit a morphological variability wider than hexaploids (Figure 1), a remarkable number of individuals morphologically overlaps with the hexaploid cytotype. Univariate analyses suggest that there is no quantitative or qualitative character allowing an unambiguous identification of cytotypes (Table 1). Based on this result, it is not possible to assign a putative ploidy level to the lectotype of *S. villosa* [26] on morphological grounds, and more in general it is not possible to study the distribution of the two cytotypes using the morphology of herbarium specimens. Therefore, albeit the tetraploid populations were detected in central-eastern Spain and the only known hexaploid population was detected in south-eastern Spain [22,27], we deem that the current shortage of chromosome data, in proportion to the wide distribution range, does not allow for speculation about a possible allopatric distribution of the two cytotypes. The absence of morphological distinctiveness between the two cytotypes agrees with their sister relationship observed in the phylogenetic tree provided by Giacò and collaborators [25]. Based on the current knowledge, the case of *S. villosa* does not fit with any of the cases presented by Soltis and collaborators [28], in which chromosome races may be worth of taxonomic distinction. Therefore, on taxonomic grounds, we deem the two cytotypes of *S. villosa* should not be recognized as distinct taxa. Indeed, also in other species of *Santolina* the co-occurrence of more than one cytotype did not lead to the recognition of separate taxa. Indeed, *S. corsica* (*S. chamaecyparissus* complex) is both tetraploid and hexaploid [23], whereas *S. montiberica* (Riv.-Guerra) R.Carbajal, L.Sáez, M.Serrano & S.Ortiz, *S. pectinata* Lag., and *S. rosmarinifolia* s.str. (*S. rosmarinifolia* complex) are both diploid and tetraploid [4,29].

The morphometric analyses carried out on all the species of *S. chamaecyparissus* complex show that the most important overall discriminant characters are those related to the leaf morphology. The length and tomentosity of leaves, as well as the number of leaf segments, their length, and how much they are spaced-out are all good discriminant characters, especially if used in combination. Conversely, the characters related to the capitulum morphology show less discriminant power. Moreover, characters such as the width of the peduncle of capitula, the shape of capitula (globose or goblet-shaped), the apex of the inter-floral bracts (rounded or truncated), and the shape of additional morphological structures on the inter-floral bracts, albeit considered important characters by some authors [3,30,31], were preliminary discarded from our analyses since they were extremely variable within the same individual.

Most species show high values of correct *a priori* classification (Table 2). The exceptions are *S. benthamiana*, *S. decumbens*, and *S. intricata*, the morphological variation of which was already discussed in detail by Giacò and collaborators [24], also in the light of their phylogenetic relations. *Santolina virens* and *S. ericoides* are morphologically close (Table 2, Figure 2), and this affinity further supports the hypothesis which sees *S. virens* as a homoploid hybrid species having *S. ericoides* and *S. rosmarinifolia* as parents [22,32]. In addition, these two putative parental taxa are sympatric in central and northern Spain, where *S. virens* is native [4]. Albeit similar, however, *S. ericoides* and *S. virens* can be easily distinguished by the shape of the leaf segment apex, that is rounded in the former and acute in the latter. A remarkable number of species is partially misclassified by Random Forest as *S. corsica* (Table 2). A possible explanation of this result lies in the high intra- and inter-populational variability documented for this species [23]. However, univariate analyses detected those morphological characters allowing an unambiguous distinction between *S. corsica* and all the other partially misclassified species. For instance, *S. vedranensis*, a narrow endemic to the islet of Es Vedrà (Balearic Islands, Spain), albeit partially misclassified with *S. corsica* (22.9%), can be easily distinguished by the degree of tomentosity of the leaves of non-flowering stems, almost glabrous in *S. vedranensis* and densely tomentose in *S. corsica*. According to Carbajal et al. [21], the taxonomic distinction of *S. vedranensis* and *S. corsica* is supported also on molecular grounds. More details regarding the characters allowing a distinction between species are provided in the identification key.

A phylogenomic analysis of the whole genus *Santolina* is currently ongoing in order to better understand the evolutionary history of species. The preliminary results [33] suggest that all the species studied here represent distinct evolutionary lineages.

In conclusion, our study filled a gap of knowledge concerning the lack of morphological diagnosability of the two cytotypes of *S. villosa* and the morphometric relations of all the species currently recognized within the *S. chamaecyparissus* complex.

## 4. Identification Key

**Figure 3:**
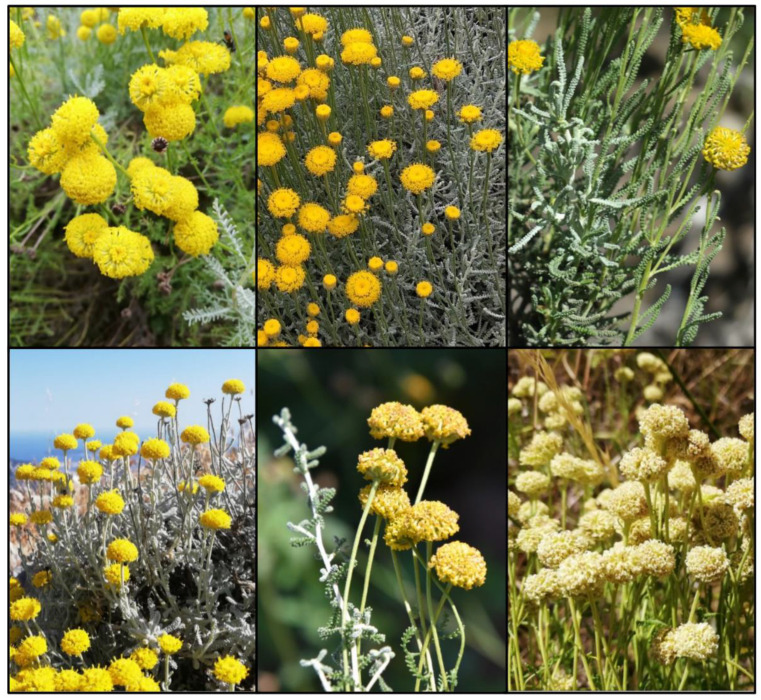

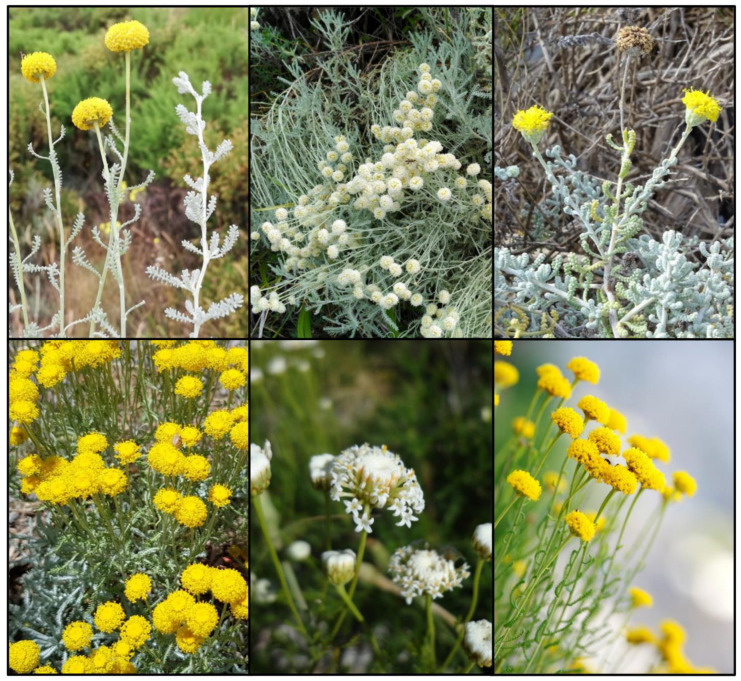
The pictures of the species of the *S. chamaecyparissus* complex. (**top**) From left to right: *Santolina benthamiana* (France, Occitanie, Prats-de-Mollo, photo by L. Peruzzi), *S. chamaecyparissus* (Italy, Tuscany, Orto e Museo Botanico di Pisa, photo by P. De Giorgi), *S. corsica* (Italy, Sardinia, Buggerru, photo by S. Cambria), *S. decumbens* s.str. (France, Provence-Alpes-Côte d’Azur, Mont Caume, photo by L. Peruzzi), *S. ericoides* (Spain, Barcelona, Sant Feliu de Codines, photo by L. Sáez), and *S. etrusca* (Italy, Tuscany, Arcidosso, photo by L. Peruzzi). (**bottom**) From left to right: *Santolina intricata* (France, Occitanie, Le Roumenga, photo by L. Peruzzi), *S. ligustica* (Italy, Liguria, Deiva Marina, photo by G. Astuti), *S. magonica* (Spain, Mallorca, Cala Mesquida, photo by L. Sáez), *S. neapolitana* (Italy, Campania, Castellammare di Stabia, photo by P. Caputo), *S. pinnata* (Italy, Tuscany, Pian della Fioba (Apuan Alps), photo by L. Peruzzi), *S. vedranensis* (Spain, Es Vedrà (Balearic Islands), and photo by J. Serapio).

For a reliable identification, complete portions composed of both flowering and non-flowering stems must be sampled. In the sampling, fragments with branched flowering stems should be preferred to fragments without branched stems. Identification must be carried out on flowering or fruiting specimens, either fresh or dry, albeit in dry specimens the color of the flowers is usually lost. In the identification process, only the longest stems, leaves, and leaf segments, and the widest capitula must be considered. It is recommended to measure the same character multiple times on distinct portions of the fragment and then to compare the mean value obtained with the variation ranges reported in the key (Table 4), instead of using a single measurement. Some parts of the identification key were taken and integrated from [24,25]. In Figure 3, photos in nature of all species, except *S. villosa* and *S. virens*, are reported.

## 5. Materials and Methods

A total amount of 27 populations was sampled in the field during the summers of 2019, 2020, and 2021. For each population, 20 flowering individuals were collected (except for *S. virens, S. chamaecyparissus*, and *S. vedranensis* for which four, nine, and 13 individuals were, respectively, sampled). Concerning Corsica and Sardinia, continental Italy, and populations from southern France and north-eastern Spain, the same individuals studied by De Giorgi and collaborators [23] and Giacò and collaborators [24,25] were analyzed. A total amount of 506 specimens was analyzed. In Table 5, information concerning all the studied populations is reported. All the studied specimens are conserved in the herbarium of Pisa (PI) (acronym follows Thiers [34]) and HD images of all of them are available at https://www.jacq.org/ (accessed on 9 November 2022).

For each individual, 31 quantitative and nine qualitative characters were measured (Table 6). All of the measurements were taken on dried material with a ruler/digital caliper or with ImageJ v.1.52b (http://rsb.info.nih.gov/ij, accessed on 30 August 2022). In this latter case, a 1200 dpi scan of the portion to measure was obtained. Tomentosity of leaves and stems was measured according to the following procedure: a portion of leaf/stem was photographed with a digital camera mounted on a stereomicroscope. Next, the area covered by tomentum was measured with ImageJ. Finally, the percentage of area covered by tomentum was calculated dividing the area covered by tomentum by the total area. The tomentosity of the non-flowering stems (ss_hair in Table 6) was transformed into an ordered factor using the following classes: 0–5% (hairless or almost hairless), 6–30% (slightly pubescent), 31–60% (pubescent), 61–90% (tomentose), and 91–100% (densely tomentose). The tomentosity of the inter-floral bracts (sq_if_hair in Table 6) was categorized based on the number of hairs: 0–3 (glabrous), 4–10 (slightly pubescent), 11–25 (pubescent), 26–50 (tomentose), and 51 or more (densely tomentose).

The morphological variation of the two cytotypes of *S. villosa* was graphically visualized with a PCoA based on Gower distance. Next, univariate analyses were conducted to check for possible morphological characters discriminant between the two cytotypes. For characters showing equal variance (Bartlett test with *p* > 0.05), a t-test was conducted. Instead, for those characters showing unequal variance (Bartlett’s test with *p* < 0.05), a Welch t-test was conducted. After that, for each significant result (Tukey-Kramer or Welch t-test with *p* < 0.05), the Cohen’s d index was calculated [35,36]. As in Giacò and collaborators [25], significant results were considered relevant only when Cohen’s d > 1.2, i.e., the two means are distant at least 1.2 standard deviations. Qualitative characters were analyzed with the Fisher’s exact test. The differences were considered significant when *p* < 0.05.

The analyses concerning the whole complex were carried out by employing a PCA based on mean values for each species. For a better visualization of the biplot, a Pearson correlation test was carried out between all pairs of variables, and highly correlated (r > 0.85) variables were discarded. Next, to check for the robustness of the morphological diagnosability of the two cytotypes of *S. villosa* and of all the species currently recognized in the *S. chamaecyparissus* complex, the Random Forest classification method (RF) was used using the R package “randomForest”, considering all species as *a priori* groups. RF was reiterated 100 times, each time half randomly splitting the dataset in the training and test subsets. Next, univariate analyses have been carried out as described above using the Hochberg’s method to adjust *p*-values and reduce the family-wise error rate. All statistical analyses were conducted in R environment [37].

## Figures and Tables

**Figure 4:**
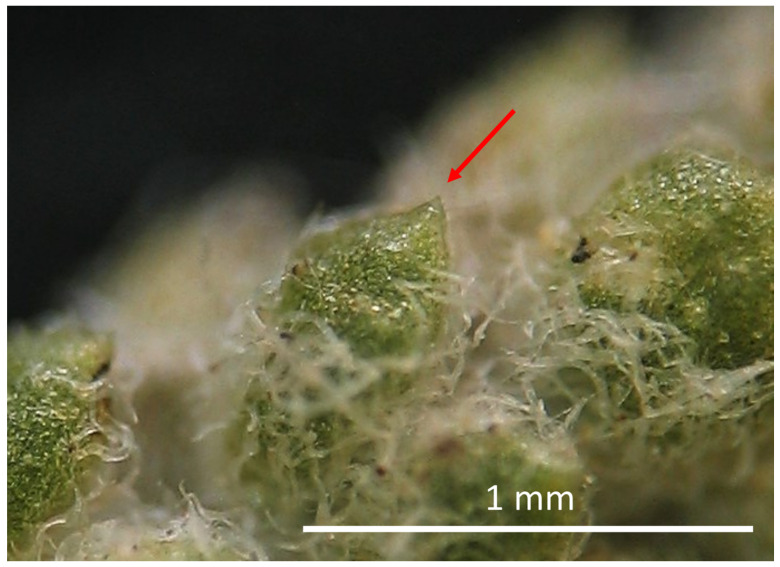
Small acute apex of leaf segments in *Santolina villosa*, indicated by the red arrow.

**Table 1:** The results of univariate analyses contrasting the two cytotypes of the polyploid Spanish endemic *Santolina villosa*. In this case, fs_len = length of the flowering stem (cm), ss_len = length of the non-flowering stem (cm), sq_if_len = length of the inter-floral bract (mm), ssl_seg_dist = distance between the segments of the non-flowering stem leaf (mm), fs_n_nodes = number of nodes of the flowering stem, ss_n_nodes = number of nodes of the non-flowering stem, ssl_hair = degree of tomentosity of the non-flowering stem leaf segment (%), and fs_hair = degree of tomentosity of the flowering stem (%).

Character	*p*-Value	Cohen’s d Value
fs_len	0.002	1.03
ss_len	0.013	0.81
sq_if_len	0.008	0.87
ssl_seg_dist	0.018	0.76
fs_n_nodes	0.013	0.81
ss_n_nodes	0.002	1.1
ssl_hair	0.024	0.73
fs_hair	0.020	0.75

**Table 2:** The confusion matrix of the Random Forest method using species of the *Santolina chamaecyparissus* complex as *a priori* groups. Values are percentages. Ben = *S. benthamiana*, Cha = *S. chamaecyparissus*, Cor = *S. corsica*, Dec = *S. decumbens*, Eri = *S. ericoides*, Etr = *S. etrusca*, Int = *S. intricata*, Lig = *S. ligustica*, Mag = *S. magonica*, Nea = *S. neapolitana*, Pin = *S. pinnata*, Ved = *S. vedranensis*, Vil = *S. villosa*, and Vir = *S. virens*.

	Ben	Cha	Cor	Dec	Eri	Etr	Int	Lig	Mag	Nea	Pin	Ved	Vil	Vir
**Ben**	94.7	0	0	2.2	0.4	0	2.3	0	0	0	0.4	0	0	0
**Cha**	0	90.0	10	0	0	0	0	0	0	0	0	0	0	0
**Cor**	0	0	99.3	0.5	0.1	0	0	0	0.1	0	0	0	0	0
**Dec**	0	0	15	81.8	0	0	2	0	1.1	0	0	0	0	0
**Eri**	0	0	0	0	100	0	0	0	0	0	0	0	0	0
**Etr**	0	0	1.1	0	0	98.9	0	0	0	0	0	0	0	0
**Int**	6.8	0	4.8	18.3	0.1	0	68.7	0	1.4	0	0	0	0	0
**Lig**	0	0	0	0.3	0	0	0.1	99.5	0	0	0	0	0	0
**Mag**	0	0	0.4	3.8	0	0	0	0	95.7	0	0	0	0.1	0
**Nea**	0	0	6.7	0	0	0.4	0.1	0	0	92.9	0	0	0	0
**Pin**	0	0	0	0	0	0	0	0	0	0	100	0	0	0
**Ved**	0	0	22.9	9	7.3	0	0	0	0.4	0	0	59.9	0.4	0
**Vil**	0	0	0	1.9	0	0	0	0	0	0	0	0	98	0
**Vir**	0	0	0	0	29.7	0	0	0	0	0	0	0	0.8	69.5

**Table 3:** The mean values ± standard deviation for each species and each quantitative character in the *Santolina chamaecyparissus* complex. For character codes see Materials and Methods. Ben = *S. benthamiana*, Cha = *S. chamaecyparissus*, Cor = *S. corsica*, Dec = *S. decumbens*, Eri = *S. ericoides*, Etr = *S. etrusca*, Int = *S. intricata*, Lig = *S. ligustica*, Mag = *S. magonica*, Nea = *S. neapolitana*, Pin = *S. pinnata*, Ved = *S. vedranensis*, Vil = *S. villosa*, and Vir = *S. virens*.

Character	Ben	Cha	Cor	Dec	Eri	Etr	Int
fs_len (cm)	22.9 ± 9.2	16.7 ± 2.6	15.2 ± 5.1	12.5 ± 5.3	19.3 ± 5.5	26.9 ± 6	21.7 ± 6.3
br_ratio	0 ± 0	0.1 ± 0.1	0.4 ± 0.3	0.1 ± 0.2	0.1 ± 0.2	0.8 ± 0.1	0.1 ± 0.2
dist_cap_lf (cm)	44.2 ± 26.7	41.9 ± 11.6	27.2 ± 16.5	16.5 ± 10.1	27.9 ± 15.3	22.1 ± 11.5	35 ± 12.1
ss_len (cm)	11.8 ± 5.2	10.4 ± 2.8	8.9 ± 3.9	8.0 ± 3.1	15.6 ± 6.5	16.8 ± 5.5	16.5 ± 6.4
cap_diam (mm)	6.6 ± 1.3	7.0 ± 0.4	6.3 ± 1.3	6.8 ± 0.9	6.8 ± 1.1	5.9 ± 0.8	6.8 ± 1.1
sq_ext_len (mm)	2.8 ± 0.6	3.2 ± 0.5	3.1 ± 0.4	2.9 ± 0.4	2.8 ± 0.4	2.9 ± 0.3	3.1 ± 0.5
sq_ext_wid (mm)	1.1 ± 0.2	1.6 ± 0.2	1.3 ± 0.2	1.2 ± 0.2	1.0 ± 0.2	1.1 ± 0.2	1.1 ± 0.2
sq_int_len (mm)	2.8 ± 0.5	3.8 ± 0.3	3.1 ± 0.4	3.1 ± 0.4	3.1 ± 0.4	3.2 ± 0.3	3.1 ± 0.4
sq_int_wid (mm)	1.3 ± 0.4	1.7 ± 0.2	1.4 ± 0.3	1.2 ± 0.2	1.3 ± 0.2	1.2 ± 0.2	1.2 ± 0.2
sq_if_len (mm)	3.0 ± 0.3	3.7 ± 0.4	3.3 ± 0.5	3.0 ± 0.3	2.9 ± 0.3	3.3 ± 0.3	3.0 ± 0.4
sq_if_wid (mm)	1.1 ± 0.2	1.2 ± 0.2	1.0 ± 0.2	1.0 ± 0.2	1.1 ± 0.2	0.9 ± 0.2	1.0 ± 0.2
flower_len (mm)	3.4 ± 0.4	4.3 ± 0.3	3.2 ± 0.4	3.3 ± 0.5	3.5 ± 0.5	3.7 ± 0.5	3.2 ± 0.4
fl_th_len (mm)	0.8 ± 0.1	0.6 ± 0.1	0.7 ± 0.1	0.7 ± 0.1	0.6 ± 0.3	1.0 ± 0.1	0.7 ± 0.1
ssl_len (mm)	26.8 ± 9.9	23.0 ± 3.4	29.8 ± 9.5	16.5 ± 6.2	11.0 ± 2.9	42.8 ± 9.9	21.3 ± 5.9
ssl_pet_len (mm)	5.7 ± 2.1	3.0 ± 0.8	3.4 ± 1.7	2.7 ± 1.6	1.1 ± 0.8	5.1 ± 2.6	4.0 ± 2.3
ssl_seg_len (mm)	4.2 ± 1.5	1.5 ± 0.2	1.3 ± 0.4	1.2 ± 0.6	1.3 ± 0.5	2.4 ± 0.6	2.9 ± 1.0
ssl_seg_wid (mm)	0.5 ± 0.1	0.8 ± 0.1	0.6 ± 0.1	0.4 ± 0.1	0.4 ± 0.1	0.4 ± 0.1	0.5 ± 0.1
ssl_seg_dist (mm)	1.6 ± 0.7	0.8 ± 0.2	0.8 ± 0.4	0.6 ± 1.1	0.5 ± 0.2	1.1 ± 0.4	1.1 ± 0.3
fsl_len (mm)	21.6 ± 8.9	13.6 ± 2.2	20.8 ± 8.3	11.9 ± 4.1	11.0 ± 3.2	28.4 ± 5.4	18.9 ± 5.8
fsl_pet_len (mm)	6.4 ± 3.3	5.4 ± 1.2	3.1 ± 1.6	1.9 ± 1.1	1.2 ± 0.8	2.8 ± 1.7	3.4 ± 2.4
fsl_seg_len (mm)	2.6 ± 0.9	1.2 ± 0.2	1.0 ± 0.4	0.9 ± 0.5	1.2 ± 0.4	1.9 ± 0.4	2.3 ± 0.7
fsl_seg_wid (mm)	0.5 ± 0.1	0.6 ± 0.1	0.5 ± 0.2	0.4 ± 0.1	0.4 ± 0.1	0.3 ± 0.1	0.5 ± 0.1
fsl_seg_dist (mm)	1.6 ± 0.9	0.4 ± 0.3	0.5 ± 0.3	0.4 ± 0.3	0.6 ± 0.2	0.9 ± 0.4	1.0 ± 0.3
fs_n_br	0 ± 0	1.0 ± 1.2	2.4 ± 3.2	0.3 ± 0.8	0.3 ± 0.9	3.1 ± 1.7	0.5 ± 1.2
fs_n_nodes	21.0 ± 3.3	12.9 ± 2.5	15.6 ± 4.2	19.2 ± 5	24.5 ± 5.1	24.4 ± 2.8	22.0 ± 4.3
ss_n_nodes	20.7 ± 4.7	16.0 ± 3.2	16.2 ± 4.0	17.2 ± 4.0	26.5 ± 7.1	24.9 ± 4.8	23.0 ± 6.5
ssl_n_seg	29.7 ± 8.8	45.6 ± 6.4	99.9 ± 32.2	61.4 ± 14.1	38.4 ± 9.6	95.8 ± 20.0	41.5 ± 8.9
fsl_n_seg	19.4 ± 5.4	14.3 ± 3.5	69.6 ± 29.2	39.4 ± 9.3	34.7 ± 8.9	67.8 ± 12.4	31.8 ± 8.0
ssl_hair	0.5 ± 0.2	0.9 ± 0.1	0.8 ± 0.2	0.8 ± 0.2	0.2 ± 0.2	0.5 ± 0.2	0.8 ± 0.1
fsl_hair	0.2 ± 0.2	0.4 ± 0.1	0.4 ± 0.2	0.5 ± 0.3	0.1 ± 0.1	0.1 ± 0.2	0.7 ± 0.2
fs_hair	0.2 ± 0.1	0.2 ± 0.1	0.4 ± 0.2	0.7 ± 0.2	0.3 ± 0.2	0.4 ± 0.2	0.6 ± 0.1
fsl_seg_ratio	5.8 ± 1.6	2.0 ± 0.3	2.0 ± 0.7	2.5 ± 1.1	3.3 ± 1.1	5.8 ± 1.6	4.5 ± 1.6
ssl_seg_ratio	8.6 ± 2.7	2.1 ± 0.4	2.4 ± 0.9	2.9 ± 1.6	3.4 ± 1.2	6.3 ± 1.7	5.8 ± 2.0
**Character**	**Lig**	**Mag**	**Nea**	**Pin**	**Ved**	**Vil**	**Vir**
fs_len (cm)	17.9 ± 4.6	10.4 ± 2.7	20.1 ± 3.4	23.3 ± 4.6	10.8 ± 3.5	16.1 ± 4.1	21.4 ± 4.2
br_ratio	0.7 ± 0.2	0.4 ± 0.3	0.5 ± 0.3	0.4 ± 0.2	0 ± 0.1	0.1 ± 0.1	0.2 ± 0.2
dist_cap_lf (cm)	16.0 ± 8.9	12.1 ± 6.2	27.1 ± 12.5	65.4 ± 26.0	21.1 ± 10.0	31.3 ± 14	61.2 ± 24.5
ss_len (cm)	10.6 ± 4.1	7.9 ± 2.7	8.7 ± 3.4	6.4 ± 3.9	6.2 ± 3.3	8.9 ± 3.6	9.0 ± 3.8
cap_diam (mm)	4.0 ± 0.6	5.3 ± 0.7	6.2 ± 1.0	6.1 ± 1.3	6.9 ± 1.2	7.3 ± 0.8	6.8 ± 1.1
sq_ext_len (mm)	2.7 ± 0.5	2.8 ± 0.5	2.7 ± 0.4	3.1 ± 0.4	3.4 ± 0.5	3.0 ± 0.5	3.2 ± 0.7
sq_ext_wid (mm)	0.9 ± 0.1	1.2 ± 0.3	1.1 ± 0.3	1.2 ± 0.1	1.5 ± 0.5	1.2 ± 0.3	1.5 ± 0.2
sq_int_len (mm)	2.6 ± 0.3	3.2 ± 0.4	2.8 ± 0.3	3.4 ± 0.5	3.8 ± 0.7	3.8 ± 0.4	3.2 ± 0.1
sq_int_wid (mm)	0.9 ± 0.1	1.6 ± 0.2	1.1 ± 0.2	1.2 ± 0.2	1.4 ± 0.2	1.5 ± 0.2	1.2 ± 0.3
sq_if_len (mm)	2.6 ± 0.3	2.7 ± 0.3	2.7 ± 0.2	3.1 ± 0.3	3.2 ± 0.4	3.4 ± 0.4	3.0 ± 0.1
sq_if_wid (mm)	0.8 ± 0.1	1.2 ± 0.2	1.0 ± 0.2	1.0 ± 0.2	1.2 ± 0.3	1.2 ± 0.2	0.9 ± 0.3
flower_len (mm)	2.6 ± 0.3	3.1 ± 0.5	3.0 ± 0.4	3.5 ± 0.6	3.7 ± 0.5	3.3 ± 0.4	2.9 ± 0.7
fl_th_len (mm)	0.8 ± 0.1	0.7 ± 0.1	1.0 ± 0.1	1.0 ± 0.2	0.7 ± 0.1	0.6 ± 0.1	0.5 ± 0.1
ssl_len (mm)	34.0 ± 9.2	10 ± 2.6	43.8 ± 6.3	41.9 ± 8.7	20.4 ± 7.4	11.8 ± 2.5	14.6 ± 2.2
ssl_pet_len (mm)	7.6 ± 2.2	1.2 ± 0.6	6.7 ± 2.4	6.3 ± 2.5	1.0 ± 1.0	1.4 ± 0.9	3.1 ± 1.4
ssl_seg_len (mm)	3.0 ± 0.9	0.9 ± 0.4	3.7 ± 1.2	4.6 ± 1.3	0.8 ± 0.2	0.8 ± 0.3	0.8 ± 0.3
ssl_seg_wid (mm)	0.4 ± 0.1	0.5 ± 0.1	0.4 ± 0.1	0.5 ± 0.1	0.4 ± 0.1	0.5 ± 0.1	0.4 ± 0.1
ssl_seg_dist (mm)	1.2 ± 0.5	0.3 ± 0.2	1.1 ± 0.5	1.4 ± 0.7	0.5 ± 0.3	0.3 ± 0.2	0.8 ± 0.1
fsl_len (mm)	22.3 ± 5.8	8.3 ± 1.7	29.1 ± 5.7	30.5 ± 5.4	16.6 ± 8.2	12.1 ± 2.2	13.2 ± 0.8
fsl_pet_len (mm)	5.6 ± 2.5	1.2 ± 0.6	4.4 ± 2.4	5.1 ± 2.1	0.7 ± 0.8	1.0 ± 0.8	2.0 ± 0.9
fsl_seg_len (mm)	2.3 ± 0.7	0.8 ± 0.3	2.6 ± 0.7	3.7 ± 0.6	0.6 ± 0.3	0.8 ± 0.2	0.6 ± 0.2
fsl_seg_wid (mm)	0.4 ± 0.1	0.4 ± 0.1	0.4 ± 0.1	0.4 ± 0.1	0.4 ± 0.1	0.4 ± 0.1	0.4 ± 0.1
fsl_seg_dist (mm)	1.2 ± 0.3	0.3 ± 0.2	0.9 ± 0.4	1.3 ± 0.4	0.6 ± 0.4	0.4 ± 0.1	0.6 ± 0.1
fs_n_br	4.2 ± 3.5	1.9 ± 1.9	2.8 ± 2.1	1.8 ± 1.4	0.2 ± 0.6	0.4 ± 1	2 ± 2.4
fs_n_nodes	20.2 ± 3.4	18.8 ± 3.6	16.7 ± 2.8	18.8 ± 3.6	16.8 ± 4.5	17.2 ± 2	25.2 ± 1.5
ss_n_nodes	20.0 ± 3.9	18.6 ± 3.8	17.1 ± 4.1	20 ± 6	17.8 ± 7.0	15.2 ± 2.9	19.2 ± 4.6
ssl_n_seg	53.5 ± 10.8	45.6 ± 11.8	83.2 ± 16.1	52.3 ± 13.5	73.6 ± 17.4	48.3 ± 11.2	33.5 ± 10.9
fsl_n_seg	33 ± 8.9	40.3 ± 12.3	60.2 ± 13.5	34.5 ± 9.0	59.8 ± 20.0	48.3 ± 9.4	38.5 ± 9.6
ssl_hair	0.8 ± 0.1	0.7 ± 0.2	0.8 ± 0.1	0 ± 0	0 ± 0	0.4 ± 0.2	0 ± 0
fsl_hair	0.7 ± 0.2	0.6 ± 0.3	0.6 ± 0.3	0 ± 0	0 ± 0	0.3 ± 0.2	0 ± 0
fs_hair	0.9 ± 0.1	0.7 ± 0.2	0.7 ± 0.2	0 ± 0	0.3 ± 0.2	0.7 ± 0.1	0 ± 0
fsl_seg_ratio	5.9 ± 2.2	2 ± 0.6	6.7 ± 1.7	9.3 ± 2.2	1.6 ± 0.8	1.8 ± 0.6	1.5 ± 0.6
ssl_seg_ratio	7.6 ± 2.2	1.9 ± 0.6	8.9 ± 3.3	9.7 ± 2.2	1.7 ± 0.6	1.8 ± 0.7	2.3 ± 1.3

**Table 4:** Identification key for all the taxa of the *Santolina chamaecyparissus* complex.

1a	Leaves of the non-flowering stems green, glabrous or scarcely pubescent (white-tomentose, at most, only on the central axis)	2
1b	Leaves of the non-flowering stems grey or white-tomentose, both on the central axis and on the leaf segments	6
2a	Leaves of the non-flowering stems with segments >2 mm long	3
2b	Leaves of the non-flowering stems with segments <2 mm long	4
3a	Segments with acute apex. Flowers white	***S. pinnata* Viv.**
3b	Segments with rounded apex. Flowers yellow	***S. benthamiana* Jord. & Fourr.**
4a	Segments with acute apex	***S. virens* Mill.** (rarely used as ornamental)
4b	Segments with rounded apex	5
5a	Leaves of the non-flowering stems 15–30 mm long, with 50–100 segments 0.5–1 mm long	***S. vedranensis* (O.Bolòs & Vigo) L.Sáez, M.Serrano, S.Ortiz & R.Carbajal**
5b	Leaves of the non-flowering stems 5–20 mm long, with 20–60 segments 0.5–2 mm long	***S. ericoides* Poir.**
6a	Involucre 3–5 mm wide and leaf segments of the non-flowering stems 2–6 mm long. Flowers white	***S. ligustica* Arrigoni**
6b	Involucre 5–8(–10) mm wide, or involucre 3–5 mm wide coupled with leaf segments <2 mm long. Flowers yellow	7
7a	Leaves of the non-flowering stems >25 mm long and with more than 65 segments, 0.4–2 mm spaced-out	8
7b	Characters never in combination as above	10
8a	Leaf segments of the flowering stems 0.5–1.5(–2) mm long. Leaf segments of the non-flowering stems 0.5–2 mm long. Floral teeth <1 mm long	***S. corsica* Jord. & Fourr.**
8b	Leaf segments of the flowering stems 1.5–4 mm long. Leaf segments of the non-flowering stems 1.5–5(–8) mm long. Floral teeth often >1 mm long	9
9a	Flowering stems branched in the upper portion, the highest branch often at more than 3/4 of the stem. Non-flowering stems 10–30 cm long. Leaves of the flowering stems with segments 1.5–2.5 mm long. Flowers pale yellow	***S. etrusca* (Lacaita) Marchi & D’Amato**
9b	Flowering stems branched or not branched. If branched, the highest branch never at more than 3/4 of the stem. Non-flowering stems mostly shorter than 10 cm. Leaves of the flowering stems with segments 1.5–4 mm long. Flowers yellow	***S. neapolitana* Jord. & Fourr.**
10a	Leaf segments with a small acute apex (see through a magnifying glass) (Figure 4)	***S. villosa* Mill.**
10b	Leaf segments rounded at apex	11
11a	Leaves of the non-flowering stems <18 mm long and capitula not totally covered by the flowers in lateral view	***S. magonica* (O.Bolòs, Molin. & P.Monts.) Romo**
11b	Leaves of the non-flowering stems >18 mm long, or leaves of the non-flowering stems <18 mm coupled with capitula covered by the flowers in lateral view	12
12a	Leaf segments of the flowering stem >2.5 times longer than wide and leaves with ≤65 segments	13
12b	Leaf segments of the flowering stem <2.5 times longer than wide, or >2.5 but number of segments >65	14
13a	Leaves of the non-flowering stems with 25–65, 0.8–2 mm spaced-out segments. Segments of the flowering stem leaves 1.5–3 mm long, 0.5–1.5 mm spaced-out	***S. intricata* Jord. & Fourr.**
13b	Leaves of the non-flowering stems with 50–80, <1 mm spaced-out, appressed segments. Segments of the flowering stem leaves 0.5–2 mm long, 0–1.0 mm spaced-out	***S. decumbens* subsp. *diversifolia* (Jord. & Fourr.) Giacò & Peruzzi**
14a	Leaves of the non-flowering stems >20 mm long	15
14b	Leaves of the non-flowering stems <20 mm long	16
15a	Flowering stems white, tomentose as (or almost as) the non-flowering stems	***S. decumbens* Mill. subsp. *decumbens***
15b	Flowering stems green, clearly less tomentose than the non-flowering stems	***S. decumbens* subsp. *tisoniana* Giacò & Peruzzi**
16a	Tubular portion of the flowers usually <4 mm long. Leaves of the flowering stems with 20–150 segments, 10–45 mm long. Pollen vital	***S. corsica* Jord. & Fourr.**
16b	Tubular portion of the flowers usually >4 mm long. Leaves of the flowering stems with 10–20(–40) segments, 10–20 mm long. Pollen aborted.	***S. chamaecyparissus* L.** (widely used as ornamental)

**Table 5:** The information concerning the populations of the *Santolina chamaecyparissus* complex analyzed in this study.

Species	N	Population	Vouchers
*S. benthamiana*	20	France, Occitanie, Prats-de-Mollo-la-Preste [WGS84: 42.407222 N, 2.523055 E]	*A. Giacò, L. Peruzzi*, 29 June 2020, PI 043080–043098, [25]
*S. chamaecyparissus*	9	France, Provence-Alpes-Côte d’Azur, Le Luc [WGS84: 43.354166 N, 6.412222 E]	*A. Giacò, L. Peruzzi*, 30 June 2020, PI 034970–034974, [23]
*S. corsica*	20	France, Corsica, Mont Pigno [WGS84: 42.7066667 N, 9.407777 E]	*A. Giacò, L. Peruzzi*, 7 July 2020, PI 036636–036647, [23]
*S. corsica*	20	Italy, Sardinia, Monte Albo [WGS84: 40.537853 N, 9.615131 E]	*G. Calvia* et al., 19 June 2020, PI 036122–036136, [23]
*S. corsica*	20	Italy, Sardinia, Buggerru [WGS84: 39.393611 N, 8.391666 E]	*G. Bacchetta* et al., 14 June 2020, PI 036613–036625, [23]
*S. corsica*	20	Italy, Sardinia, San Benedetto (Iglesias) [WGS84: 39.360311 N, 8.558333 E]	*G. Bacchetta* et al., 14 June 2020, PI 036068–036085, [23]
*S. corsica*	20	Italy, Sardinia, Laconi [WGS84: 39.847483 N, 9.071944 E]	*G. Bacchetta* et al., 15 June 2020, PI 036052–036067, [23]
*S. corsica*	20	Italy, Sardinia, Monte Spada [WGS84: 40.058586 N, 9.293333 E]	*G. Bacchetta* et al., 14 June 2020, PI 036106–036121, [23]
*S. corsica*	20	Italy, Sardinia, Monte Corrasi [WGS84: 40.256878 N, 9.426253 E]	*G. Bacchetta* et al., 14 June 2020, PI 036648–036663, [23]
*S. decumbens* (subsp. *decumbens*)	20	France, Provence-Alpes-Côte d’Azur, Mont Caume [WGS84: 43.184768 N, 5.908187 E]	*A. Giacò, L. Peruzzi*, 27 June 2020, PI 043107–043118, [25]
*S. decumbens* (subsp. *diversifolia*)	20	France, Provence-Alpes-Côte d’Azur, Sisteron [WGS84: 44.153341 N, 5.953744 E]	*A. Giacò, L. Peruzzi*, 11 July 2021, PI 053348–053364, [25]
*S. decumbens* (subsp. *tisoniana*)	20	France, Provence-Alpes-Côte d’Azur, La Fare-les-Oliviers [WGS84: 43.539610 N, 5.172029]	*A. Giacò, L. Peruzzi*, 28 June 2020, PI 043099–043106, [25]
*S. ericoides*	20	France, Occitanie, Béziers [WGS84: 43.28959 N 3.18539 E]	*A. Giacò, L. Peruzzi*, 28 June 2020, PI 036086–036100, [25]
*S. ericoides*	20	Spain, Barcelona province, Sant Feliu de Codines [WGS84: 41.692294 N, 2.174761 E]	*L. Sáez*, 7 July 2020, PI 043077, PI 057135–057154, [25]
*S. ericoides*	20	Spain, Lleida province, Torà [WGS84: 41.814325 N, 1.404588 E]	*L. Sáez*, 13 July 2020, PI 043076, PI 057115–057134, [25]
*S. etrusca*	20	Italy, Tuscany, Radicofani [WGS84: 42.954283 N, 11.778340 E]	*G. Astuti, P. De Giorgi*, 14 July 2020, PI 040480–040501, [24]
*S. etrusca*	20	Italy, Lazio, Bassano in Teverina [WGS84: 42.487438 N, 12.327856 E]	*G. Astuti, P. De Giorgi*, 14 July 2020, PI 040468–040479, [24]
*S. intricata*	20	France, Occitanie, Montalba-le-Château, Le Roumenga [WGS84: 42.699054 N, 2.552235 E]	*A. Giacò, L. Peruzzi*, 28 June 2020, PI 043079, PI 057098–057114, [25]
*S. ligustica*	20	Italy, La Spezia, Levanto [WGS84: 44.230000 N, 9.589120 E]	*G. Astuti, S. Chiletti*, 22 July 2019, PI 030947–030971, [24]
*S. magonica*	20	Spain, Balearic Islands, Menorca, Cala Tirant [WGS84: 40.045132 N, 4.102162 E]	*P. Fraga,* 9 July 2020, PI 043078, PI 056632–056651
*S. magonica*	20	Spain, Balearic Islands, Mallorca, Cala Mesquida [WGS84: 39.7458333 N, 3.4319444 E]	*E. Guasp,* 28 June 2020, PI 043119–043127
*S. neapolitana*	20	Italy, Campania, Castellammare di Stabia [WGS84: 40.658447 N, 14.498790 E]	*P. Caputo, D. De Luca*, 7 August 2020, PI 040502–040521, [24]
*S. pinnata*	20	Italy, Tuscany, Apuan Alps, Forno [WGS84: 44.084178 N, 10.183817 E]	*G. Astuti, P. De Giorgi*, 9 July 2020, PI 040442–040461, [24]
*S. vedranensis*	13	Spain, Balearic Islands, Es Vedrà [WGS84: 38.867298 N, 1.196176 E]	*J. Serapio,* 20 July 2020, PI 043075–057906
*S. villosa* (4*x*)	20	Spain, Madrid, Arganda del Rey [WGS84: 40.332155 N, 3.435883 W]	*M. Serrano,* 10 August 2021, PI 053328–053347
*S. villosa* (6*x*)	20	Spain, Granada, Gor [WGS84: 37.403440 N, 3.009413 W]	*R. Carballal,* 10 July 2021, PI 056652–056672
*S. virens*	4	Spain, Burgos, Fuentenebro [WGS84: 41.516782 N, 3.756607 W]	*M. Serrano,* 16 June 2021, PI 049949–049952

**Table 6:** The morphometric characters analyzed in the *Santolina chamaecyparissus* complex. QC = quantitative continuous, QD = quantitative discrete, CO = ordered factor, CN = nominal, CB = binary.

Code	Description of the Character	Type	Tool
Vegetative Parts			
fs_len	Length of the flowering stem (cm)	QC	Ruler
br_ratio	Ratio between the highest ramification of the flowering stem and fs_len	QC	Ruler
dist_cap_lf	Distance between the highest leaf on the stem and the floral head (mm)	QC	Caliper
fs_n_br	Number of branches of the flowering stem	QD	
br_type	Type of branch (no branch/parallel/or erect-patent)	CN	
fs_n_nodes	Number of nodes of the flowering stem	QD	
ss_len	Length of the non-flowering stem (cm)	QC	Ruler
ss_n_nodes	Number of nodes of the non-flowering stem	QD	
ss_hair	Tomentosity of the non-flowering stem (hairless/slightly pubescent/pubescent/hairy/densely hairy)	CO	ImageJ
fs_hair	Degree of tomentosity of the flowering stem (%)	QC	ImageJ
fsl_n_seg	Number of segments on the flowering stem leaf (the longest)	QD	
ssl_n_seg	Number of segments on the non-flowering stem leaf (the longest)	QD	
ssl_len	Length of the non-flowering stem leaf (mm)	QC	ImageJ
ssl_pet_len	Length of the petiole of the non-flowering stem leaf (mm)	QC	ImageJ
ssl_seg_len	Length of the segment of the non-flowering stem leaf (mm)	QC	ImageJ
ssl_seg_dist	Distance between the segments of the non-flowering stem leaf (mm)	QC	ImageJ
ssl_seg_type	Segment of the non-flowering stem pointed at apex (Yes/No)	CB	
fsl_len	Length of the flowering stem leaf (mm)	QC	ImageJ
fsl_pet_len	Length of the petiole of the flowering stem leaf (mm)	QC	ImageJ
fsl_seg_len	Length of the segment of the flowering stem leaf (mm)	QC	ImageJ
fsl_seg_dist	Distance between the segments of the flowering stem leaf (mm)	QC	ImageJ
fsl_seg_type	Segment of the flowering stem pointed at apex (Yes/No)	CB	
ssl_hair	Degree of tomentosity of the non-flowering stem leaf segment (%)	QC	ImageJ
fsl_hair	Degree of tomentosity of the flowering stem leaf segment (%)	QC	ImageJ
fsl_seg_ratio	Ratio between the length and the width of the segment of the flowering stem leaf		
ssl_seg_ratio	Ratio between the length and the width of the segment of the non-flowering stem leaf		
**Floral Head**			
cap_diam	Diameter of the floral head involucre (mm)	QC	Caliper
fl_col	Colour of the flowers (white/pale yellow/yellow)	CO	
fl_type	Flowers totally covering the involucre (Yes/No)	CB	
flower_len	Length of the floral tube (mm)	QC	ImageJ
fl_th_len	Length of the floral tooth (mm)	QC	ImageJ
sq_ext_len	Length of the external involucral bract (mm)	QC	ImageJ
sq_ext_wid	Width of the external involucral bract (mm)	QC	ImageJ
sq_int_len	Length of the internal involucral bract (mm)	QC	ImageJ
sq_int_wid	Width of the internal involucral bract (mm)	QC	ImageJ
sq_if_len	Length of the inter-floral bract (mm)	QC	ImageJ
sq_if_wid	Width of the inter-floral bract (mm)	QC	ImageJ
sq_if_n_hair	Tomentosity of the inter-floral bract (hairless/slightly pubescent/pubescent/hairy/densely hairy)	CO	ImageJ
sq_ext_hair	Tomentosity of the external involucral bract (hairless/only on the margin/everywhere)	CO	ImageJ
sq_int_hair	Tomentosity of the internal involucral bract (hairless/only on the margin/everywhere)	CO	ImageJ

## Data Availability

Not applicable.

## Supplementary Materials

The following supporting information can be downloaded at: https://www.mdpi.com/article/10.3390/plants11243458/s1. Table S1: Mean values ± standard deviation for each character and for each studied population in the *Santolina chamaecyparissus* complex. Table S2: Significantly different morphological character-states in the *Santolina chamaecyparissus* complex. Table S3: Significantly different morphological character-states in the *Santolina chamaecyparissus* complex.
