# Supplementary material for: A Morphometric Analysis of the Santolina chamaecyparissus Complex (Asteraceae)
Source: Plants (Basel). 2022 Dec 9;11(24):3458. doi: 10.3390/plants11243458 (PMC9785004; doi:10.3390/plants11243458)
Supplement: Supplementary file 1 [file plants-11-03458-s001.zip › plants-2069814-supplementary.pdf]

Table S1. Mean values ± standard deviation for each studied population and each character in the *Santolina chamaecyparissus* complex. Character code follow Table 6. Ben-LC = *S. benthamiana* (Prats-de-Mollo-la-Preste), Cha-luc = *S. chamaecyparissus* (Le Luc), Cor-alb = *S. corsica* (Monte Albo), Cor-bug = *S. corsica* (Buggerru), Cor-gen = *S. corsica* (Monte Spada), Cor-lac = *S. corsica* (Laconi), Cor-ben = *S. corsica* (San Benedetto), Cor-oli = *S. corsica* (Monte Corrasi), Dec-dec = *S. decumbens* subsp. *decumbens* (Mont Caume), Dec-sis = *S. decumbens* subsp. *diversifolia* (Sisteron), Dec-tis = *S. decumbens* subsp. *tisoniana* (la-Fare-les-Oliviers), Eri-LC = *S. ericoides* (Béziers), Eri-sfc = *S. ericoides* (Sant Feliu de Codines), Eri-tor = *S. ericoides* (Torà), Etr-Bas = *S. etrusca* (Bassano in Teverina), Etr-LC = *S. etrusca* (Radicofani), Int-rou = *S. intricata* (Le Roumenga), Lig-LC = *S. ligustica* (Levanto), Mag-LC = *S. magonica* (Cala Tirant), Mag-mes = *S. magonica* (Cala Mesquida), Nea-LC = *S. neapolitana* (Castellammare di Stabia), Pin-LC = *S. pinnata* (Forno), Ved-LC = *S. vedranensis* (Es Vedrà), Vil-arg = *S. villosa* (Arganda del Rey), Vil-gor = *S. villosa* (Las Viñas), Vir-fue = *S. virens* (Fuentenebro).

	**Ben-LC**	**Cha-luc**	**Cor-alb**	**Cor-LC**	**Cor-bug**	**Cor-gen**	**Cor-lac**	**Cor-ben**	**Cor-oli**
fs_len	22.9 ± 9.2	16.7 ± 2.6	15.7 ± 3.5	12.6 ± 3.9	11.2 ± 4.4	15.7 ± 4.9	16.4 ± 3.8	20.4 ± 5.2	14.2 ± 4.7
br_ratio	0 ± 0	0.1 ± 0.1	0.2 ± 0.3	0.6 ± 0.2	0.7 ± 0.3	0.1 ± 0.2	0.3 ± 0.3	0.6 ± 0.3	0.1 ± 0.2
dist_cap_lf	44.2 ± 26.7	41.9 ± 11.6	24.7 ± 12.4	20.4 ± 7.3	11.5 ± 6.4	36.4 ± 22.5	27.5 ± 11.6	34 ± 13.8	35.9 ± 19.2
ss_len	11.8 ± 5.2	10.4 ± 2.8	9.3 ± 2.2	6.7 ± 3.6	5.9 ± 3.2	10.2 ± 3.7	10.9 ± 2.9	11.8 ± 3.8	7 ± 3.2
cap_diam	6.6 ± 1.3	7 ± 0.4	7.1 ± 1.6	5.1 ± 0.7	5.6 ± 0.8	6.6 ± 1.1	6.8 ± 1.3	6.8 ± 1.1	6.5 ± 0.9
sq_ext_len	2.8 ± 0.6	3.2 ± 0.5	3 ± 0.4	2.9 ± 0.4	3 ± 0.4	3.1 ± 0.5	3 ± 0.3	3.2 ± 0.4	3.2 ± 0.4
sq_ext_wid	1.1 ± 0.2	1.6 ± 0.2	1.3 ± 0.2	1.2 ± 0.2	1.3 ± 0.2	1.4 ± 0.2	1.3 ± 0.3	1.3 ± 0.3	1.3 ± 0.2
sq_int_len	2.8 ± 0.5	3.8 ± 0.3	3.1 ± 0.4	2.9 ± 0.3	3 ± 0.4	3.5 ± 0.4	3 ± 0.4	3.2 ± 0.3	3.2 ± 0.6
sq_int_wid	1.3 ± 0.4	1.7 ± 0.2	1.4 ± 0.2	1.2 ± 0.1	1.2 ± 0.1	1.5 ± 0.4	1.4 ± 0.3	1.5 ± 0.2	1.4 ± 0.2
sq_if_len	3 ± 0.3	3.7 ± 0.4	3.4 ± 0.5	3.1 ± 0.4	2.9 ± 0.3	3.4 ± 0.5	3.1 ± 0.3	3.3 ± 0.3	3.9 ± 0.5
sq_if_wid	1.1 ± 0.2	1.2 ± 0.2	1 ± 0.2	0.9 ± 0.2	1 ± 0.2	1.1 ± 0.2	1 ± 0.2	1 ± 0.2	1 ± 0.3
fl_len	3.4 ± 0.4	4.3 ± 0.3	3.3 ± 0.4	2.8 ± 0.3	3.1 ± 0.3	3.3 ± 0.4	2.9 ± 0.5	3.3 ± 0.4	3.5 ± 0.3
fl_th_len	0.8 ± 0.1	0.6 ± 0.1	0.8 ± 0.1	0.7 ± 0.1	0.6 ± 0.1	0.7 ± 0.1	0.7 ± 0.2	0.7 ± 0.1	0.8 ± 0.1
ssl_len	26.8 ± 9.9	23 ± 3.4	36.5 ± 10.4	33.1 ± 9.3	24.9 ± 8.3	23.9 ± 3.5	25.1 ± 4.5	38.5 ± 8.8	26.2 ± 6.8
ssl_pet_len	5.7 ± 2.1	3 ± 0.8	4.3 ± 2.2	3.2 ± 1.6	3.3 ± 1.5	2.2 ± 1	3.2 ± 1.8	4.5 ± 1.8	2.9 ± 1
ssl_seg_len	4.2 ± 1.5	1.5 ± 0.2	1.2 ± 0.5	1.5 ± 0.3	1 ± 0.4	1.3 ± 0.3	1.3 ± 0.4	1.4 ± 0.2	1.3 ± 0.3
ssl_seg_dist	1.6 ± 0.7	0.8 ± 0.2	0.9 ± 0.4	0.9 ± 0.4	0.4 ± 0.3	0.8 ± 0.3	0.7 ± 0.3	0.7 ± 0.4	0.9 ± 0.4
fsl_len	21.6 ± 8.9	13.6 ± 2.2	21.8 ± 8.2	22.4 ± 5.5	21.8 ± 8.7	16.3 ± 4.1	15.8 ± 5.3	31.1 ± 8.5	16.1 ± 4.5
fsl_petiole_len	6.4 ± 3.3	5.4 ± 1.2	4 ± 1.6	2.3 ± 1	2.5 ± 1.1	2.9 ± 1.2	2.9 ± 1.7	4.8 ± 1.5	2.4 ± 1.2
fsl_seg_len	2.6 ± 0.9	1.2 ± 0.2	0.7 ± 0.3	1.3 ± 0.4	0.8 ± 0.2	1.1 ± 0.4	1 ± 0.4	1.1 ± 0.3	0.9 ± 0.2
fsl_seg_dist	1.6 ± 0.9	0.4 ± 0.3	0.7 ± 0.4	0.7 ± 0.2	0.4 ± 0.3	0.3 ± 0.3	0.3 ± 0.3	0.6 ± 0.3	0.6 ± 0.3
fs_n_br	0 ± 0	1 ± 1.2	0.7 ± 1.4	4 ± 2.9	5 ± 5.3	1 ± 1.6	1.5 ± 1.6	4 ± 2.8	0.3 ± 0.7
fs_n_nodes	21 ± 3.3	12.9 ± 2.5	14.8 ± 2.5	15 ± 2.4	19.9 ± 6	14.3 ± 2.9	16.3 ± 3.5	17.2 ± 3.9	11.5 ± 2.2
ss_n_nodes	20.7 ± 4.7	16 ± 3.2	17.1 ± 3.2	15.4 ± 3.1	16 ± 5.2	16.5 ± 3.3	17.1 ± 4.8	18.4 ± 3.1	12.7 ± 2.8
ssl_n_seg	29.7 ± 8.8	45.6 ± 6.4	102.8 ± 20.9	135.8 ± 37.7	121.6 ± 31.3	72.4 ± 12	73.5 ± 16.8	109.5 ± 18.7	83.7 ± 14.8
fsl_n_seg	19.4 ± 5.4	14.3 ± 3.5	59.7 ± 17.3	90.2 ± 28.6	105.2 ± 32.1	48.8 ± 9.2	48.6 ± 15	82.2 ± 15.4	52.4 ± 16.4
ssl_hair	0.5 ± 0.2	0.9 ± 0.1	0.7 ± 0.2	0.8 ± 0.1	0.8 ± 0.2	0.7 ± 0.2	0.6 ± 0.2	0.8 ± 0.1	0.9 ± 0.1
fsl_hair	0.2 ± 0.2	0.4 ± 0.1	0.4 ± 0.2	0.7 ± 0.2	0.6 ± 0.2	0.2 ± 0.1	0.3 ± 0.1	0.5 ± 0.1	0.5 ± 0.2
fs_hair	0.2 ± 0.1	0.2 ± 0.1	0.3 ± 0.2	0.5 ± 0.1	0.7 ± 0.2	0.3 ± 0.1	0.3 ± 0.1	0.4 ± 0.2	0.3 ± 0.1
fsl_seg_ratio	5.8 ± 1.6	2 ± 0.3	1.6 ± 0.6	3.1 ± 0.8	1.5 ± 0.3	1.8 ± 0.6	2 ± 0.6	2 ± 0.4	1.9 ± 0.5
ssl_seg_ratio	8.6 ± 2.7	2.1 ± 0.4	2.3 ± 1.1	3.6 ± 0.9	1.8 ± 0.4	2.1 ± 0.4	2.2 ± 0.9	2.3 ± 0.7	2.3 ± 0.6

	**Dec-dec**	**Dec-div**	**Dec-tis**	**Eri-LC**	**Eri-sfc**	**Eri-tor**	**Etr-bas**	**Etr-LC**	**Int-rou**
fs_len	10.8 ± 2.6	18.1 ± 4.4	8.6 ± 2.9	18.6 ± 6.7	18.6 ± 4.1	20.8 ± 5.1	23.1 ± 4	30.6 ± 5.3	21.7 ± 6.3
br_ratio	0.1 ± 0.1	0.1 ± 0.2	0 ± 0	0.2 ± 0.3	0 ± 0	0 ± 0.1	0.8 ± 0.1	0.8 ± 0.1	0.1 ± 0.2
dist_cap_lf	15.3 ± 7.4	23.7 ± 11.8	10.6 ± 5.4	20.4 ± 10.4	29.8 ± 15.7	33.4 ± 16.5	17.5 ± 6.2	26.7 ± 13.7	35 ± 12.1
ss_len	6.3 ± 2.5	10.1 ± 3	7.7 ± 2.6	13.7 ± 6.8	15.3 ± 4.2	17.9 ± 7.5	13.8 ± 4.7	19.9 ± 4.6	16.5 ± 6.4
cap_diam	6.9 ± 0.7	7 ± 0.9	6.4 ± 1	6.2 ± 1.3	7.2 ± 0.8	7 ± 0.9	6.2 ± 0.8	5.7 ± 0.7	6.8 ± 1.1
sq_ext_len	2.8 ± 0.4	3.2 ± 0.4	2.7 ± 0.4	2.8 ± 0.3	2.8 ± 0.4	3 ± 0.4	2.9 ± 0.2	2.9 ± 0.3	3.1 ± 0.5
sq_ext_wid	1.1 ± 0.2	1.2 ± 0.2	1.1 ± 0.2	1.2 ± 0.2	1 ± 0.2	1 ± 0.2	1.1 ± 0.2	1 ± 0.2	1.1 ± 0.2
sq_int_len	3.1 ± 0.4	3.4 ± 0.4	2.9 ± 0.3	3 ± 0.4	3.2 ± 0.3	3.1 ± 0.4	3.3 ± 0.2	3.2 ± 0.3	3.1 ± 0.4
sq_int_wid	1.2 ± 0.2	1.3 ± 0.2	1.1 ± 0.2	1.3 ± 0.2	1.3 ± 0.2	1.3 ± 0.1	1.2 ± 0.2	1.2 ± 0.2	1.2 ± 0.2
sq_if_len	3 ± 0.3	3.1 ± 0.2	2.8 ± 0.3	2.9 ± 0.3	3 ± 0.2	2.7 ± 0.2	3.3 ± 0.3	3.2 ± 0.4	3 ± 0.4
sq_if_wid	1 ± 0.2	1.1 ± 0.2	1 ± 0.2	1.1 ± 0.2	1.2 ± 0.2	1.1 ± 0.2	0.8 ± 0.1	0.9 ± 0.2	1 ± 0.2
fl_len	3 ± 0.4	3.3 ± 0.5	3.5 ± 0.5	3.4 ± 0.6	3.5 ± 0.6	3.7 ± 0.4	3.7 ± 0.6	3.7 ± 0.4	3.2 ± 0.4
fl_th_len	0.7 ± 0.1	0.7 ± 0.1	0.7 ± 0.1	0.5 ± 0.1	0.7 ± 0.5	0.6 ± 0.1	1 ± 0.1	0.9 ± 0.1	0.7 ± 0.1
ssl_len	14.6 ± 3.8	23.4 ± 4.1	11.4 ± 2.2	11 ± 3.1	11.4 ± 2.1	10.6 ± 3.5	38.2 ± 8.9	47.5 ± 8.7	21.3 ± 5.9

ssl_pet_len	2.9 ± 1.2	3.7 ± 1.8	1.3 ± 0.7	1 ± 0.8	1.6 ± 0.8	0.8 ± 0.4	5.1 ± 2.8	5.1 ± 2.4	4 ± 2.3
ssl_seg_len	0.9 ± 0.2	2 ± 0.4	0.8 ± 0.2	0.9 ± 0.4	1.5 ± 0.4	1.4 ± 0.5	2.2 ± 0.5	2.6 ± 0.6	2.9 ± 1
ssl_seg_dist	0.3 ± 0.3	1 ± 1.8	0.3 ± 0.2	0.5 ± 0.2	0.5 ± 0.3	0.6 ± 0.2	0.9 ± 0.3	1.2 ± 0.5	1.1 ± 0.3
fsl_len	12.3 ± 3.2	15.4 ± 3.1	8 ± 1.9	10.7 ± 3.8	12.1 ± 2.2	10.3 ± 3.2	26.3 ± 5.4	30.4 ± 4.7	18.9 ± 5.8
fsl_pet_len	2.1 ± 0.9	2.3 ± 1.2	1.1 ± 0.6	0.9 ± 0.6	1.8 ± 0.8	0.8 ± 0.5	2.7 ± 1.4	2.9 ± 1.9	3.4 ± 2.4
fsl_seg_len	0.8 ± 0.3	1.5 ± 0.4	0.6 ± 0.1	0.9 ± 0.3	1.3 ± 0.4	1.4 ± 0.2	1.8 ± 0.4	2 ± 0.3	2.3 ± 0.7
fsl_seg_dist	0.3 ± 0.3	0.6 ± 0.2	0.3 ± 0.1	0.6 ± 0.3	0.6 ± 0.2	0.6 ± 0.2	0.8 ± 0.3	1 ± 0.4	1 ± 0.3
fs_n_br	0.5 ± 1	0.3 ± 0.9	0 ± 0	0.8 ± 1.4	0 ± 0	0.1 ± 0.4	3.6 ± 2	2.6 ± 1.3	0.5 ± 1.2
fs_n_nodes	19.8 ± 3.5	19.4 ± 3.6	18.4 ± 7.2	24.9 ± 6.6	26.2 ± 3.7	22.4 ± 4	24.6 ± 2.8	24.2 ± 2.7	22 ± 4.3
ss_n_nodes	14.8 ± 3.4	16.8 ± 2.7	20 ± 4.1	24.6 ± 7.7	28 ± 5.9	26.8 ± 7.3	24.7 ± 4.7	25.1 ± 5	23 ± 6.5
ssl_n_seg	60.5 ± 12.4	61.9 ± 16.6	61.8 ± 13.7	46.7 ± 10.6	35.4 ± 6.6	33.2 ± 4.3	98.4 ± 22	93.2 ± 17.9	41.5 ± 8.9
fsl_n_seg	40.4 ± 9.4	39 ± 9.7	38.8 ± 9.1	40.5 ± 9.2	31.9 ± 8.3	31.6 ± 6.2	68.8 ± 13.6	66.8 ± 11.2	31.8 ± 8
ssl_hair	0.9 ± 0.1	0.8 ± 0.1	0.6 ± 0.1	0.2 ± 0.1	0.2 ± 0.1	0.1 ± 0.2	0.5 ± 0.2	0.4 ± 0.3	0.8 ± 0.1
fsl_hair	0.9 ± 0.1	0.4 ± 0.2	0.3 ± 0.2	0.2 ± 0.1	0.2 ± 0.1	0.1 ± 0.1	0.2 ± 0.2	0.1 ± 0.1	0.7 ± 0.2
fs_hair	0.9 ± 0.1	0.6 ± 0.2	0.6 ± 0.1	0.3 ± 0.1	0.3 ± 0.2	0.4 ± 0.2	0.5 ± 0.2	0.3 ± 0.1	0.6 ± 0.1
fsl_seg_ratio	1.8 ± 0.6	3.7 ± 0.9	1.8 ± 0.3	2.8 ± 0.8	3.3 ± 1.1	3.7 ± 1.3	5.7 ± 1.6	5.8 ± 1.7	4.5 ± 1.6
ssl_seg_ratio	1.9 ± 0.6	4.8 ± 1.3	2.1 ± 0.5	2.7 ± 1	3.6 ± 0.9	3.9 ± 1.3	6.1 ± 1.7	6.6 ± 1.8	5.8 ± 2

	**Lig-LC**	**Mag-mes**	**Mag-LC**	**Nea-LC**	**Pin-LC**	**Ved-LC**	**Vil-arg**	**Vil-gor**	**Vir-fue**
fs_len	17.9 ± 4.6	9 ± 1.6	11.8 ± 2.8	20.1 ± 3.4	23.3 ± 4.6	10.8 ± 3.5	18.1 ± 4.3	14.2 ± 2.9	21.4 ± 4.2
br_ratio	0.7 ± 0.2	0.2 ± 0.3	0.6 ± 0.2	0.5 ± 0.3	0.4 ± 0.2	0 ± 0.1	0.1 ± 0.2	0 ± 0	0.2 ± 0.2
dist_cap_lf	16 ± 8.9	9.6 ± 4.8	14.6 ± 6.6	27.1 ± 12.5	65.4 ± 26	21.1 ± 10	32.3 ± 16.4	30.4 ± 11.6	61.2 ± 24.5
ss_len	10.6 ± 4.1	6.2 ± 1.5	9.6 ± 2.6	8.7 ± 3.4	6.4 ± 3.9	6.2 ± 3.3	10.3 ± 3.9	7.5 ± 2.7	9 ± 3.8
cap_diam	4 ± 0.6	5.2 ± 0.6	5.4 ± 0.8	6.2 ± 1	6.1 ± 1.3	6.9 ± 1.2	7.3 ± 0.9	7.3 ± 0.8	6.8 ± 1.1
sq_ext_len	2.7 ± 0.5	2.5 ± 0.4	3.1 ± 0.4	2.7 ± 0.4	3.1 ± 0.4	3.4 ± 0.5	3 ± 0.6	3 ± 0.5	3.2 ± 0.7
sq_ext_wid	0.9 ± 0.1	1.2 ± 0.3	1.2 ± 0.2	1.1 ± 0.3	1.2 ± 0.1	1.5 ± 0.5	1.2 ± 0.3	1.1 ± 0.3	1.5 ± 0.2
sq_int_len	2.6 ± 0.3	3 ± 0.3	3.4 ± 0.4	2.8 ± 0.3	3.4 ± 0.5	3.8 ± 0.7	3.9 ± 0.4	3.7 ± 0.5	3.2 ± 0.1
sq_int_wid	0.9 ± 0.1	1.6 ± 0.2	1.5 ± 0.2	1.1 ± 0.2	1.2 ± 0.2	1.4 ± 0.2	1.5 ± 0.2	1.5 ± 0.2	1.2 ± 0.3
sq_if_len	2.6 ± 0.3	2.7 ± 0.3	2.8 ± 0.2	2.7 ± 0.2	3.1 ± 0.3	3.2 ± 0.4	3.2 ± 0.4	3.5 ± 0.3	3 ± 0.1
sq_if_wid	0.8 ± 0.1	1.1 ± 0.2	1.2 ± 0.2	1 ± 0.2	1 ± 0.2	1.2 ± 0.3	1.3 ± 0.2	1.2 ± 0.2	0.9 ± 0.3
fl_len	2.6 ± 0.3	2.9 ± 0.5	3.4 ± 0.3	3 ± 0.4	3.5 ± 0.6	3.7 ± 0.5	3.3 ± 0.3	3.4 ± 0.4	2.9 ± 0.7
fl_th_len	0.8 ± 0.1	0.7 ± 0.1	0.7 ± 0.1	1 ± 0.1	1 ± 0.2	0.7 ± 0.1	0.6 ± 0.1	0.6 ± 0.1	0.5 ± 0.1
ssl_len	34 ± 9.2	9.2 ± 2.1	10.8 ± 2.8	43.8 ± 6.3	41.9 ± 8.7	20.4 ± 7.4	12.1 ± 2.4	11.4 ± 2.6	14.6 ± 2.2
ssl_pet_len	7.6 ± 2.2	1.2 ± 0.7	1.1 ± 0.6	6.7 ± 2.4	6.3 ± 2.5	1 ± 1	1.3 ± 0.8	1.5 ± 1	3.1 ± 1.4
ssl_seg_len	3 ± 0.9	0.8 ± 0.2	1.1 ± 0.5	3.7 ± 1.2	4.6 ± 1.3	0.8 ± 0.2	0.8 ± 0.3	0.8 ± 0.3	0.8 ± 0.3
ssl_seg_dist	1.2 ± 0.5	0.3 ± 0.2	0.3 ± 0.2	1.1 ± 0.5	1.4 ± 0.7	0.5 ± 0.3	0.4 ± 0.2	0.3 ± 0.1	0.8 ± 0.1
fsl_len	22.3 ± 5.8	7.8 ± 1.3	8.9 ± 1.9	29.1 ± 5.7	30.5 ± 5.4	16.6 ± 8.2	12.6 ± 2.5	11.6 ± 1.7	13.2 ± 0.8
fsl_pet_len	5.6 ± 2.5	1.1 ± 0.5	1.2 ± 0.7	4.4 ± 2.4	5.1 ± 2.1	0.7 ± 0.8	0.8 ± 0.8	1.2 ± 0.7	2 ± 0.9
fsl_seg_len	2.3 ± 0.7	0.7 ± 0.1	1 ± 0.3	2.6 ± 0.7	3.7 ± 0.6	0.6 ± 0.3	0.8 ± 0.2	0.7 ± 0.3	0.6 ± 0.2
fsl_seg_dist	1.2 ± 0.3	0.3 ± 0.1	0.4 ± 0.2	0.9 ± 0.4	1.3 ± 0.4	0.6 ± 0.4	0.4 ± 0.1	0.3 ± 0.1	0.6 ± 0.1
fs_n_br	4.2 ± 3.5	1 ± 1.5	2.8 ± 2	2.8 ± 2.1	1.8 ± 1.4	0.2 ± 0.6	0.9 ± 1.2	0 ± 0	2 ± 2.4
fs_n_nodes	20.2 ± 3.4	20.5 ± 3.2	17.1 ± 3.2	16.7 ± 2.8	18.8 ± 3.6	16.8 ± 4.5	18 ± 1.7	16.4 ± 2	25.2 ± 1.5
ss_n_nodes	20 ± 3.9	18.5 ± 3.3	18.6 ± 4.4	17.1 ± 4.1	20 ± 6	17.8 ± 7	16.8 ± 2.7	13.8 ± 2.2	19.2 ± 4.6
ssl_n_seg	53.5 ± 10.8	44.1 ± 11.4	47 ± 12.3	83.2 ± 16.1	52.3 ± 13.5	73.6 ± 17.4	47.8 ± 8.6	48.9 ± 13.4	33.5 ± 10.9
fsl_n_seg	33 ± 8.9	39.7 ± 10.4	41 ± 14.1	60.2 ± 13.5	34.5 ± 9	59.8 ± 20	48 ± 8.6	48.6 ± 10.3	38.5 ± 9.6
ssl_hair	0.8 ± 0.1	0.9 ± 0.1	0.6 ± 0.2	0.8 ± 0.1	0 ± 0	0 ± 0	0.3 ± 0.2	0.5 ± 0.2	0 ± 0
fsl_hair	0.7 ± 0.2	0.8 ± 0.2	0.4 ± 0.2	0.6 ± 0.3	0 ± 0	0 ± 0	0.3 ± 0.2	0.4 ± 0.1	0 ± 0
fs_hair	0.9 ± 0.1	0.8 ± 0.1	0.5 ± 0.2	0.7 ± 0.2	0 ± 0	0.3 ± 0.2	0.7 ± 0.1	0.6 ± 0.1	0 ± 0
fsl_seg_ratio	5.9 ± 2.2	1.6 ± 0.3	2.3 ± 0.6	6.7 ± 1.7	9.3 ± 2.2	1.6 ± 0.8	1.9 ± 0.5	1.6 ± 0.6	1.5 ± 0.6
ssl_seg_ratio	7.6 ± 2.2	1.7 ± 0.3	2.2 ± 0.7	8.9 ± 3.3	9.7 ± 2.2	1.7 ± 0.6	2 ± 0.8	1.5 ± 0.6	2.3 ± 1.3

Table S2. Significantly different morphological differences in the *Santolina chamaecyparissus* complex. In the lower triangle, the number of quantitative characters showing Cohen's d > 1.2 is reported. In the higher triangle, the number of qualitative characters is reported. Ben = *S. benthamiana,* Cha = *S. chamaecyparissus,* Cor = *S. corsica,* Dec = *S. decumbens,* Eri = *S. ericoides,* Etr = *S. etrusca,* Int = *S. intricata,* Lig = *S. ligustica,* Mag = *S. magonica,* Nea = *S. neapolitana,* Pin = *S. pinnata,* Ved = *S. vedranensis,* Vil = *S. villosa,* Vir = *S. virens*.

	**Ben**	**Cha**	**Cor**	**Dec**	**Eri**	**Etr**	**Int**	**Lig**	**Mag**	**Nea**	**Pin**	**Ved**	**Vil**	**Vir**
**Ben**	-	4	6	4	5	8	2	6	6	8	6	3	7	7
**Cha**	16	-	1	4	6	5	2	3	1	3	7	3	2	5
**Cor**	10	7	-	4	6	7	3	5	4	4	7	4	6	5
**Dec**	12	11	5	-	3	6	2	4	4	6	8	3	5	6
**Eri**	12	15	12	7	-	8	3	6	6	8	8	5	7	4
**Etr**	8	26	12	17	15	-	7	3	5	5	6	8	4	7
**Int**	5	15	10	10	11	8	-	4	4	6	7	3	5	6
**Lig**	10	20	17	17	20	15	11	-	3	5	6	6	5	8
**Mag**	16	19	12	5	13	22	14	18	-	4	8	4	3	6
**Nea**	9	17	10	12	18	11	8	7	16	-	7	6	3	3
**Pin**	9	18	16	15	17	13	12	16	18	10	-	8	6	2
**Ved**	14	10	5	6	13	15	14	22	7	19	16	-	6	6
**Vil**	17	12	12	2	12	23	17	24	7	16	17	6	-	3
**Vir**	10	8	7	4	3	10	7	14	5	12	11	3	5	-

Table S3. Significantly different morphological characters in the *Santolina chamaecyparissus* complex. In the first triangle of the table, the quantitative characters showing Cohen's d > 1.2 are reported for each pair of species. In the higher triangle, the qualitative characters for each pair of species are reported. In the following list, the number of occurrences of each variable is reported in parentheses (character codes follow Table 6: a = fs_len (42), b = dist_cap_lf (28), c = ss_len (21), d = cap_diam (21), e = sq_ext_len (4), f = sq_ext_wid (17), g = sq_int_len (32), h = sq_int_wid (27), i = sq_if_len (31), j = sq_if_wid (17), k = fl_len (25), l = fl_th_len (35), m = ssl_len (59), n = ssl_pet_len (48), o = ssl_seg_len (61), p = ssl_seg_dist (30), q = fsl_len (52), r = fsl_pet_len (42), s = fsl_seg_len (58), t = fsl_seg_dist (38), u = fs_n_nodes (37), v = ss_n_nodes (16), w = ssl_n_seg (49), x = fsl_n_seg (50), y = ssl_hair (63), z = fsl_hair (57), à = fs_hair (69), ò = fsl_seg_ratio, ù = ssl_seg_ratio, A = br_type (58), B = fl_col (35), C = sq_ext_hair (50), D = sq_int_hair (70), E = sq_if_n_hair (41), F = ss_hair (53), F = fl_type (55), H = ssl_seg_type (51), J = fsl_seg_type (47).

	Ben	Cha	Cor	Dec	Eri	Etr	Int	Lig	Mag	Nea	Pin	Ved	Vil	Vir
**Ben**		A, D, F, F	A, C, D, F, F, H	C, D, F, H	C, D, E, F, H	A, B, C, D, E, F, H, J	D, F	A, B, C, D, E, F	A, C, D, F, F, H	A, C, D, E, F, F, H, J	A, B, D, F, H, J	C, F, F	A, C, D, F, F, H, J	A, C, D, F, F, H, J
**Cha**	f, g, h, i, k, n, o, p, q, s, t, u, w, y, ò, ù		F	A, D, E, F	A, C, D, E, F, F	A, B, F, H, J	D, F	A, B, F	A	F, H, J	B, C, D, F, F, H, J	A, C, D	H, J	C, D, F, H, J
**Cor**	n, o, s, u, w, x, y, à, ò, ù	g, h, k, q, r, w, x		A, D, E, F	A, C, D, E, F, F	A, B, D, E, F, H, J	A, F, F	A, B, D, E, F	A, D, E, F	D, E, H, J	B, C, D, F, F, H, J	A, C, D, F	A, D, E, F, H, J	C, D, F, H, J
**Dec**	n, o, r, s, t, w, x, y, z, à, ò, ù	b, f, g, h, i, k, r, u, x, y, à	m, q, w, x, à		C, D, F	A, B, D, E, H, J	E, F	A, B, D, E	A, D, E, F	A, D, E, F, H, J	A, B, C, D, E, F, H, J	C, D, F	D, E, F, H, J	C, D, F, F, H, J
**Eri**	m, n, o, p, q, r, s, x, y, à, ò, ù	f, g, h, i, k, m, n, r, u, v, x, y, z, ò, ù	i, m, n, q, r, u, v, w, x, y, z, ò	a, c, v, w, y, z, à		A, B, C, D, E, F, H, J	C, D, E	A, B, C, D, E, F	A, C, D, E, F, F	A, C, D, E, F, F, H, J	A, B, C, D, E, F, H, J	C, D, E, F, F	C, D, E, F, F, H, J	F, F, H, J
**Etr**	j, l, m, o, r, w, x, à	a, b, c, d, f, g, h, j, k, l, m, n, o, q, r, s, t, u, v, w, x, y, z, à, ò, ù	a, c, l, m, o, q, s, u, v, z, ò, ù	a, c, l, m, o, q, s, t, u, v, w, x, y, z, à, ò, ù	a, i, j, l, m, n, o, p, q, s, w, x, y, ò, ù		A, B, D, E, F, H, J	B, H, J	A, B, F, H, J	A, B, F, H, J	A, B, C, D, E, F	A, B, C, D, E, F, H, J	A, B, E, F	A, B, C, D, E, F, F
**Int**	w, x, y, z, à	f, g, h, i, k, o, s, t, u, x, y, z, à, ò, ù	a, o, s, t, u, w, x, z, ò, ù	a, b, c, o, q, s, t, w, ò, ù	m, n, o, p, q, s, t, y, z, à, ù	l, m, q, w, x, y, z, à		A, B, D, F	A, D, F, F	A, D, E, F, H, J	A, B, C, D, F, H, J	C, D, F	D, F, F, H, J	C, D, F, F, H, J
**Lig**	b, d, i, j, k, w, x, y, z, à	b, d, f, g, h, i, j, k, m, n, o, q, s, t, u, x, z, à, ò, ù	d, f, g, h, i, k, n, o, r, s, t, w, x, z, à, ò, ù	d, f, g, h, i, j, k, m, n, o, q, r, s, t, à, ò, ù	d, g, h, j, k, m, n, o, p, q, r, s, t, v, w, y, z, à, ò, ù	a, c, d, g, h, i, k, l, r, u, w, x, y, z, à	b, d, f, g, h, i, j, k, m, n, à		A, B, F	B, E, F, H, J	C, D, E, F, H, J	A, B, C, D, E, F	A, B, F, H, J	B, C, D, E, F, F, H, J
**Mag**	a, b, m, n, o, p, q, r, s, t, w, x, z, à, ò, ù	a, b, d, f, g, i, k, m, n, o, p, q, r, s, u, x, y, z, à	a, b, d, i, m, n, p, q, r, w, x, à	d, h, m, n, q	a, b, c, d, h, t, u, v, y, z, à, ò, ù	a, c, h, i, j, l, m, n, o, p, q, r, s, t, u, v, w, x, z, à, ò, ù	a, b, c, d, h, m, n, o, p, q, s, t, ò, ù	a, d, f, g, h, j, k, m, n, o, p, q, r, s, t, à, ò, ù		A, F, H, J	A, B, C, D, F, F, H, J	A, C, D, F	A, H, J	A, C, D, F, H, J
**Nea**	i, l, m, u, w, x, y, z, à	f, g, h, i, k, l, m, n, o, q, s, t, w, x, à, ò, ù	a, i, l, m, n, o, s, à, ò, ù	a, l, m, n, o, q, s, t, w, x, ò, ù	c, l, m, n, o, p, q, r, s, u, v, w, x, y, z, à, ò, ù	a, c, g, i, k, o, u, v, y, z, à	c, l, m, q, u, w, x, ò	d, j, l, m, w, x, à	a, b, h, l, m, n, o, p, q, r, s, t, w, x, ò, ù		B, C, D, E, F, H, J	A, C, D, E, H, J	A, E, F	C, D, F
**Pin**	l, m, s, w, x, y, z, à, ò	a, f, h, k, l, m, n, o, q, s, t, u, x, y, z, à, ò, ù	a, b, l, m, n, o, q, s, t, w, x, y, z, à, ò, ù	a, b, l, m, n, o, q, r, s, t, y, z, à, ò, ù	b, c, l, m, n, o, p, q, r, s, t, w, y, z, à, ò, ù	b, c, o, r, s, u, w, x, y, z, à, ò, ù	b, c, l, m, o, q, s, y, z, à, ò, ù	b, d, f, g, h, i, j, k, l, o, q, s, y, z, à, ò	a, b, h, i, l, m, n, o, p, q, r, s, t, y, z, à, ò, ù	b, g, i, s, w, x, y, z, à, ò		A, B, C, D, F, F, H, J	A, B, C, D, F, F	B, F
**Ved**	a, g, n, o, p, r, s, t, w, x, y, z, ò, ù	a, b, n, o, r, s, w, x, y, z	n, o, r, y, z	n, o, y, z, à, ù	a, c, e, m, o, s, u, w, x, y, z, ò, ù	a, c, e, l, m, n, o, p, q, r, s, u, y, ò, ù	a, c, n, o, p, r, s, w, x, y, z, à, ò, ù	a, d, e, f, g, h, i, j, k, m, n, o, p, r, s, t, x, y, z, à, ò, ù	d, i, m, w, y, z, à	a, e, g, h, i, k, l, m, n, o, p, q, r, s, y, z, à, ò, ù	a, b, l, m, n, o, p, q, r, s, t, w, x, à, ò, ù		A, C, D, F, H, J	A, C, D, F, H, J

**Vil**	g, l, m, n, o, p, q, r, s, t, u, v, w, x, à, ò, ù	f, k, m, n, o, p, r, s, u, x, y, à	g, j, m, n, o, p, q, r, w, x, y, à	g, y	c, g, i, o, s, t, u, v, x, à, ò, ù	a, c, d, g, h, j, l, m, n, o, p, q, r, s, t, u, v, w, x, z, à, ò, ù	c, g, j, m, n, o, p, q, s, t, u, v, x, y, z, ò, ù	b, d, f, g, h, i, j, k, l, m, n, o, p, q, r, s, t, v, x, y, z, à, ò, ù	a, b, d, g, i, q, y	g, h, i, l, m, n, o, p, q, r, s, t, w, y, ò, ù	a, b, l, m, n, o, p, q, r, s, t, x, y, z, à, ò, ù	a, m, w, y, z, à		C, D, F
**Vir**	m, o, p, q, r, s, y, à, ò, ù	i, k, m, r, u, y, z, à	m, q, u, w, y, z, à	u, y, z, à	y, z, à	k, l, m, o, q, s, w, y, à, ò	o, s, t, y, z, à, ò	g, i, m, o, q, r, s, t, u, y, z, à, ò, ù	q, u, y, z, à	l, m, o, q, s, u, w, y, z, à, ò, ù	l, m, o, q, r, s, t, u, à, ò, ù	u, w, à	g, u, y, z, à	
